# Dengue virus compartmentalization during antibody-enhanced infection

**DOI:** 10.1038/srep40923

**Published:** 2017-01-13

**Authors:** Eugenia Z. Ong, Summer L. Zhang, Hwee Cheng Tan, Esther S. Gan, Kuan Rong Chan, Eng Eong Ooi

**Affiliations:** 1 Experimental Therapeutics Centre, Agency for Science, Technology and Research (A*STAR), 138669, Singapore; 2 Program in Emerging Infectious Diseases, Duke-NUS Medical School, 169857, Singapore; 3 Department of Microbiology and Immunology, National University of Singapore, 8 Medical Drive, Block MD4, 117545, Singapore; 4 Saw Swee Hock School of Public Health, National University of Singapore, 12 Science Drive 2, 117597, Singapore; 5 Singapore MIT Alliance Research and Technology, Infectious Diseases Interdisciplinary Research Group, CREATE Campus, 138602, Singapore

## Abstract

Secondary infection with a heterologous dengue virus (DENV) serotype increases the risk of severe dengue, through a process termed antibody-dependent enhancement (ADE). During ADE, DENV is opsonized with non- or sub-neutralizing antibody levels that augment entry into monocytes and dendritic cells through Fc-gamma receptors (FcγRs). We previously reported that co-ligation of leukocyte immunoglobulin-like receptor-B1 (LILRB1) by antibody-opsonized DENV led to recruitment of SH2 domain-containing phosphatase-1 (SHP-1) to dephosphorylate spleen tyrosine kinase (Syk) and reduce interferon stimulated gene induction. Here, we show that LILRB1 also signals through SHP-1 to attenuate the otherwise rapid acidification for lysosomal enzyme activation following FcγR-mediated uptake of DENV. Reduced or slower trafficking of antibody-opsonized DENV to lytic phagolysosomal compartments, demonstrates how co-ligation of LILRB1 also permits DENV to overcome a cell-autonomous immune response, enhancing intracellular survival of DENV. Our findings provide insights on how antiviral drugs that modify phagosome acidification should be used for viruses such as DENV.

Over the last decade alone, the global burden of dengue has doubled, with an estimated 50 to 100 million symptomatic cases annually^1^. The risk of severe disease is augmented when dengue virus (DENV) is opsonized with non- or sub-neutralizing levels of antibodies that ligate Fc-gamma receptors (FcγRs) for enhanced entry and replication in monocytes, macrophages and dendritic cells. This phenomenon, termed antibody-dependent enhancement (ADE), engenders the elevated viraemia and vascular leakage that is characteristic of severe dengue. Indeed, we have recently demonstrated ADE clinically and identified how this process could be exploited to enhance live attenuated viral vaccination^2^.

ADE modifies DENV entry into target cells through FcγR. However, activation of various receptor-mediated pathways, such as downregulation of intracellular innate and adaptive antiviral mechanisms, could also contribute to enhanced dengue pathogenesis^3^. Activating FcγRs are known to signal through phosphorylation of immunoreceptor tyrosine-based activation motif (ITAM) and spleen tyrosine kinase (Syk)^4^. Activated Syk controls a number of pathways, including actin remodelling necessary for phagocytosis^5^ and STAT-1-dependent interferon-stimulated gene (ISG) induction independent of interferon receptor signaling^4^. Induction of ISGs would create an intracellular environment unfavorable for enhanced DENV replication. To counter this early activating FcγR-triggered antiviral response, DENV co-ligates the inhibitory leukocyte immunoglobulin-like receptor B1 (LILRB1) during ADE^6^. LILRB1 recruits SH2 domain-containing phosphatase-1 (SHP-1) to dephosphorylate Syk and downregulate STAT-1 induction of ISG response^6^,^7^.

Besides inhibiting early ISG induction, it is also possible that DENV alters cellular compartmentalization, which is a cell-autonomous immune response^8^, for enhanced replication by co-ligating LILRB1. This is especially since the ITAM-Syk signaling axis also governs FcγR-mediated phagocytosis^5^. Previous studies focusing on cellular uptake of DENV were performed on epithelial cell lines such as C6/36, Vero, BS-C-1 and Huh-7^9^,^10^,^11^,^12^, that collectively showed that DENV enters the host cell via clathrin-mediated endocytosis. DENV is then trafficked in Rab5 (early) and Rab7 (late) endosomes, before undergoing fusion in acidic late endosomes. However, DENV uptake and trafficking routes may vary according to type of host cell and the type of receptor used for entry^9^,^10^,^11^,^12^. Moreover, our understanding of DENV trafficking following FcγR-mediated uptake, and the implication of how FcγR signaling through ITAM and Syk could modulate trafficking and enhance viral replication during ADE remains nascent. Here, we show that LILRB1 signaling directs DENV-containing phagosomes into less acidified compartments that prevent rapid lysosomal degradation of DENV. Likewise, inhibition of phagosomal acidification by lysosomotropic drugs also led to increased antibody-dependent infection, suggesting caution on using such drugs for anti-dengue therapy.

## Results

### Isolation and characterization of DENV endocytic vesicles

We had previously obtained two subclones from limiting dilution of THP-1 cells, namely THP-1.2R (ADE-resistant) and THP-1.2S (ADE-susceptible)^6^. While both subclones supported similar levels of DENV uptake and infection under DENV only conditions, infection under ADE conditions led to significantly higher DENV-2 titres in THP-1.2S compared to THP-1.2R. The difference in susceptibility to ADE was due to higher levels of LILRB1 expression on THP-1.2S. Antibody-opsonized DENV co-ligated LILRB1 to down-regulate activating FcγR-mediated signaling, reducing induction of ISGs for enhanced DENV replication in THP-1.2S^6^. Since LILRB1 signaling modulates Syk activity, which in turn regulates phagosome trafficking and maturation^13^, we examined here if reduced Syk activity results in altered phagosomal compartmentalization of DENV that could also contribute to enhanced viral replication during ADE.

**Figure 1:**
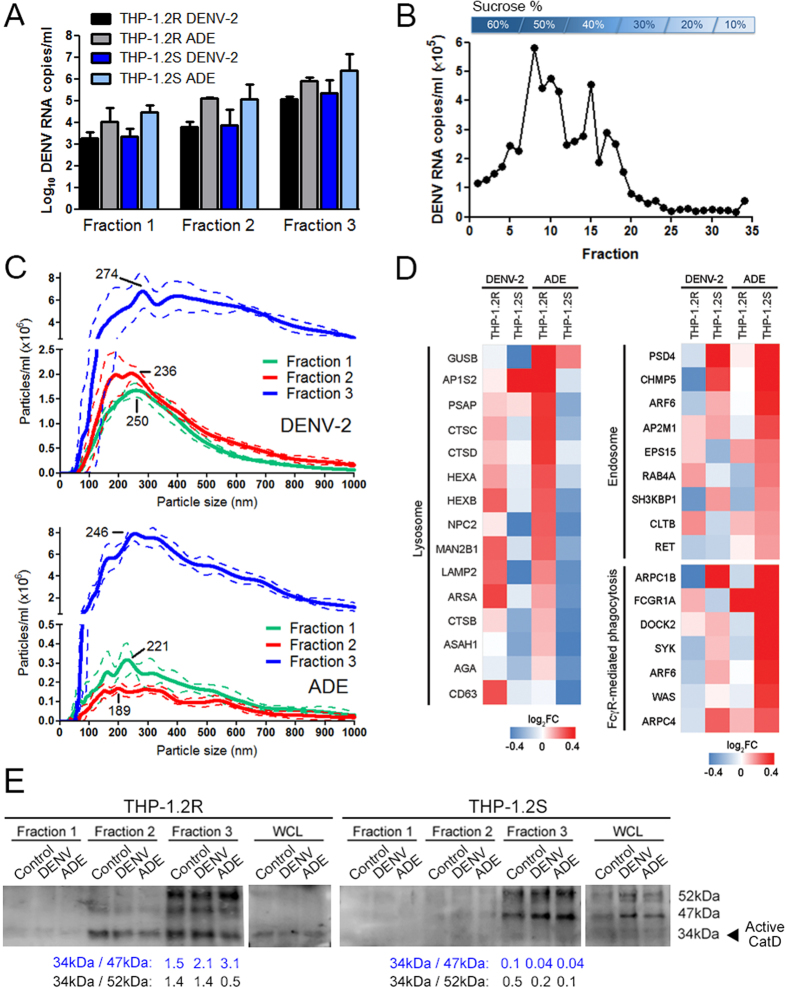
Isolation of DENV endocytic vesicles reveals DENV compartmentalization during ADE. (**A**) Viral RNA copy numbers recovered from step sucrose gradient purified fractions collected from THP-1.2R or THP-1.2S at 2 hours post-infection (hpi) under DENV-2 or h3H5-opsonized DENV-2 infection (ADE). (**B**) Viral RNA copy numbers recovered from continuous sucrose gradient purified fractions collected from THP-1.2R at 2 hpi under ADE conditions. Fractions indicated are correlated to their corresponding sucrose concentrations. (**C**) Concentration and modal size (numbers indicated) of DiD-labelled nanoparticles from fractions collected from THP-1.2S at 2 hpi under DENV-2 or ADE conditions. Solid line indicates mean, and dotted lines indicate s.d. (**D**) Heat map showing log_2_ fold change of proteins (fraction 3) enriched in THP-1.2R and THP-1.2S at 2 hpi under DENV-2 or ADE conditions, relative to uninfected cells. (**E**) Western blot showing higher levels of active CatD (34 kDa) in fraction 3 of THP-1.2R compared to THP-1.2S, in cells that were infected under DENV only or ADE conditions, or uninfected control cells. Whole cell lysate (WCL) was obtained before ultracentrifugation of cell lysates to obtain subcellular fractions. Numbers below Western blot indicate active CatD levels relative to CatD precursor proteins (47 kDa and 52 kDa) in fraction 3. Data expressed as mean ± s.d. from three independent experiments.

To investigate how compartmentalization was modified, we adapted a protocol previously used for purification of latex bead-containing phagosomes on a step sucrose gradient^14^ to isolate DENV endocytic vesicles following infection of THP-1 subclones under DENV only or ADE conditions (Supplementary Fig. S1). Recovery of DENV RNA was most abundant in fraction 3, or the densest fraction collected (Fig. 1A). Similar findings were obtained when DENV endocytic vesicles were isolated using a continuous sucrose gradient, which allows flotation of DENV-containing vesicles at their buoyant density. Importantly, the peak in viral RNA recovery using both methods of isolation corresponded to fractions of similar density, reinforcing the reproducibility of this assay (Fig. 1B). As the yield of viral RNA recovery during purification of DENV endocytic vesicles with a step sucrose gradient was higher, this method was used for subsequent experiments.

Particles in isolated fractions of step sucrose gradient were further characterized using Nanosight, which enables quantification and sizing of nanoparticles. As the sizes of endosomal and lysosomal compartments range from 200 nm to 600 nm^15^,^16^, the use of Nanosight allows tracking of DENV with cellular endocytic machinery. DENV was first labeled with DiD (1,1′-dioctadecyl-3,3,3′,3′– tetramethylindodicarbocyanine, 4-chlorobenzenesulfonate salt), a lipophilic fluorescent dye. Following uptake and fusion of the virus and host cell membranes, its fluorescence unquenches, allowing discrimination of DiD-particles using Nanosight under fluorescence mode. The modal size of DiD-particles was 2 to 3 fold larger than modal size of nanoparticles detected under light scatter mode (Fig. 1C, Supplementary Fig. S1). Importantly, the majority of DiD-particles were detected in fraction 3 in both DENV only and ADE infection (Fig. 1C), consistent with the bulk of viral RNA being recovered from fraction 3 (Fig. 1A). The modal size of 274 nm and 246 nm for DiD-particles isolated following DENV only and ADE infection, respectively, also suggests the possibility that DENV is contained within endosomal vesicles (Fig. 1C).

### DENV is trafficked to lysosomal compartments upon FcγR-mediated entry

Western blot probing for the expression of endosomal trafficking markers from isolated fractions revealed lower expression of Rab-5 and EEA-1, markers for early endosomal compartments, relative to Rab-7 and LAMP-1, markers for late endosomal compartments (Supplementary Fig. S1). This suggests that at 2 hours post-infection, DENV is compartmentalized in late endosomes or lysosomes following uptake into the host cell. The enrichment of endosomal markers in isolated fractions relative to whole cell lysate is further validation that purification of DENV endocytic vesicles can be performed on a step sucrose gradient (Supplementary Fig. S1). Organelle markers were also used to verify the purity of DENV endocytic vesicle isolation. Although Golgi apparatus (GM130) and peroxisomes (PMP70) were not detected, both ER (calnexin and BiP) and mitochondria (HSP60) co-isolated with DENV endocytic vesicles (Supplementary Fig. S1). ER proteins are known to mediate phagosome trafficking and have been co-isolated with latex bead phagosomes^17^. HSP60, which could be important for DENV replication as siRNA knockdown of HSP60 in U937 monocytic cells led to reduced viral replication and increased IFN-α production^18^, was also detected in our Western blots.

To attain a more granular level of information on how DENV compartmentalization is altered during ADE, we performed isobaric tag for relative and absolute quantitation (iTRAQ) analysis on DENV endocytic vesicles isolated from fraction 3. Interestingly, lysosomal proteins were enriched in THP-1.2R but reduced in THP-1.2S during ADE (Fig. 1D). Conversely, proteins involved in endocytosis and FcγR-mediated phagocytosis were enriched in THP-1.2S during ADE (Fig. 1D). That DENV was trafficked to lysosomal compartments in THP-1.2R during ADE was substantiated by activation of lysosomal hydrolases, which are processed to their catalytically active form upon phagosome acidification. Specifically, the expression of active cathepsin D (CatD), a lysosomal protease with optimal activity at acidic pH, was higher in THP-1.2R compared to THP-1.2S (Fig. 1E).

### LILRB1 modulates DENV phagosome acidification

Enrichment of lysosomal proteins and activation of lysosomal hydrolases suggests higher levels of phagosome acidification in THP-1.2R during ADE. To test if Syk activity affects DENV phagosome acidification, a hallmark of phagosome maturation, we labeled DENV with both pH-sensitive pHrodo dye, and pH-insensitive Alexa Fluor 488 (AF488) dye to track intracellular DENV. Since acidification of the subcellular environment increases fluorescence intensity of pHrodo but not AF488, the ratio of fluorescence intensity between pHrodo and AF488 served as a semi-quantitative measure of phagosome acidification. This approach was previously used to assess phagosome acidification during infection with bacteria that was simultaneously labelled with pHrodo and a pH-insensitive fluorescent probe^19^,^20^. Only batches of DENV that showed at least 70% of co-labelling with pHrodo and AF488 (Supplementary Fig. S2) were used for subsequent experiments.

To test if pHrodo/AF488-labeled DENV would be sensitive to changes in phagosome acidification, we infected Vero cells pre-treated with different concentrations of chloroquine (CQ). As pH of intracellular compartments increased with concentration of CQ used, fluorescence intensity of pHrodo decreased and we observed reduced overlap of pHrodo and E protein (Supplementary Fig. S2). This indicates that pHrodo/AF488-labeled DENV is sensitive to changes in pH and can be used to examine semi-quantitatively differences in phagosome acidification.

**Figure 2:**
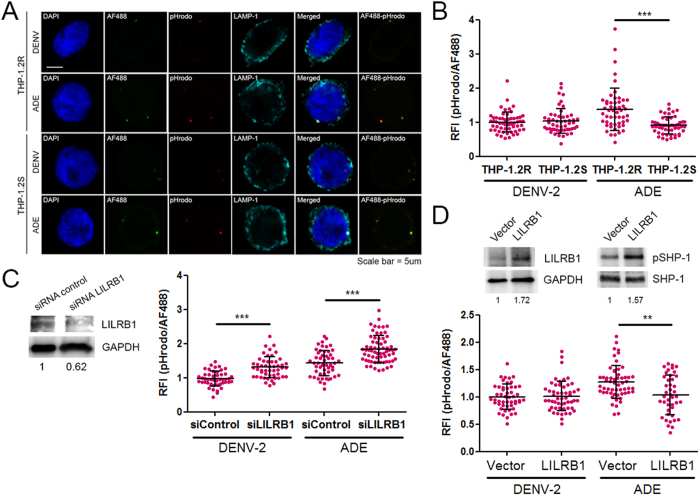
Differential phagosome acidification in THP-1 subclones is modulated by LILRB1 signaling. (**A**) Co-localization of pHrodo (red) and AF488 (green) labelled DENV-2 with LAMP-1 (cyan) in THP-1.2R and THP-1.2S at 2 hpi under DENV-2 or ADE conditions. Scale bar is 5 μm. (**B**) DENV-2 phagosome acidification expressed as relative fluorescence intensity (RFI) of pHrodo and AF488 in THP-1.2R and THP-1.2S at 2 hpi under DENV-2 or ADE conditions, normalized to DENV infection in THP-1.2R. (**C**) RFI of pHrodo and AF488 at 2 hpi after LILRB1 knockdown in THP-1.2S, normalized to DENV infection in THP-1.2S transfected with control siRNA. (**D**) RFI of pHrodo and AF488 at 2 hpi after LILRB1 overexpression in THP-1.2R, normalized to DENV infection in THP-1.2R transfected with empty vector. Numbers below Western blot indicate LILRB1 or pSHP-1 levels relative to GAPDH or SHP-1. Each dot represents a single virus particle, data expressed as mean ± s.d. from two independent experiments. ***P < 0.0001, **P < 0.01.

Use of pHrodo/AF488-labeled DENV to infect subclones under ADE conditions showed higher levels of phagosome acidification during ADE in THP-1.2R relative to THP-1.2S (Fig. 2A,B). In contrast, no difference in phagosome acidification was observed during DENV only infection in both subclones (Fig. 2A,B). Reduction in LILRB1 expression through pre-transfection with siRNA against LILRB1 in THP-1.2S resulted in increased phagosome acidification, compared to pre-transfection with scrambled control siRNA (Fig. 2C). Conversely, overexpression of LILRB1 in THP-1.2R led to an expected increase in pSHP-1 levels and reduced phagosome acidification during ADE (Fig. 2D).

**Figure 3:**
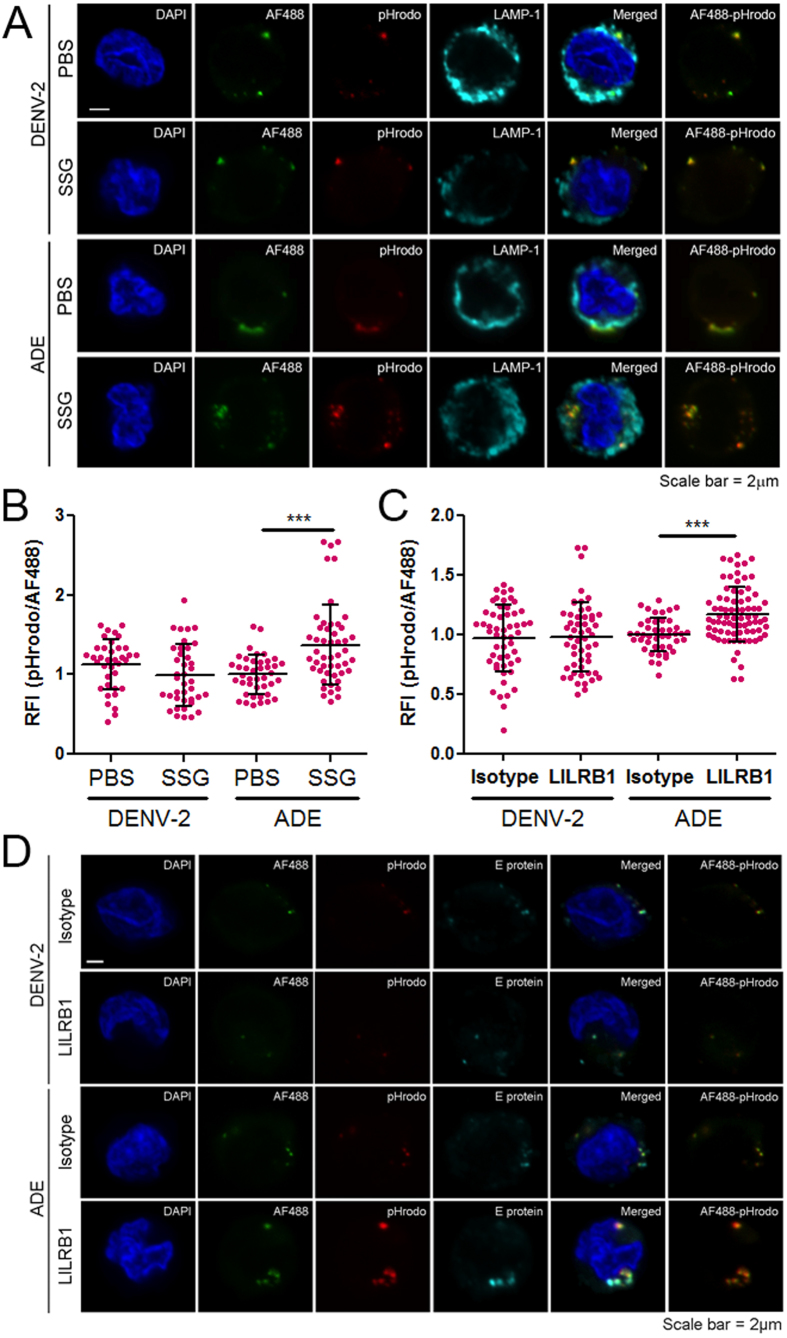
DENV phagosome acidification is reduced upon co-ligation of LILRB1 in primary monocytes. (**A**,**B**) Co-localization of pHrodo (red) and AF488 (green) labelled DENV-2 with LAMP-1 (cyan) (**A**) and RFI of pHrodo and AF488, normalized to ADE infection in PBS-treated primary monocytes (**B**) at 2 hpi in PBS- or sodium stibogluconate (SSG)-treated primary monocytes infected under DENV-2 or ADE conditions. (**C**,**D**) RFI of pHrodo and AF488, normalized to ADE infection in primary monocytes pre-treated with isotype antibody control (**C**) and co-localization of pHrodo (red) and AF488 (green) labelled DENV-2 with E protein (cyan) (**D**) at 2 hpi in primary monocytes pre-treated with isotype antibody control or polyclonal anti-LILRB1 antibody and infected under DENV-2 or ADE conditions. Scale bar is 2 μm. Each dot represents a single virus particle, data expressed as mean ± s.d. from two independent experiments. ***P < 0.0001.

Similar findings were recapitulated in primary monocytes. Increased phagosome acidification following ADE infection was observed following pre-treatment of primary monocytes with sodium stibogluconate, a SHP-1 inhibitor (Fig. 3A,B). Likewise, blocking LILRB1 with anti-LILRB1 antibodies in primary monocytes increased phagosome acidification during ADE infection (Fig. 3C,D). Collectively, our data indicates that LILRB1 signals through SHP-1 for reduced acidification in DENV phagosomes, which likely prevents DENV from being degraded by lysosomal enzymes before the virus completely uncoats.

### Inhibition of phagosome acidification enhances DENV replication

DENV is an enveloped virus that depends on low pH as a catalyst for E protein trimerization, which is thought to be the conformation necessary for membrane fusion and release of its genome into the cytosol for replication^21^. However, FcγR-mediated uptake of antibody-opsonized DENV during ADE also leads to rapid DENV phagosome acidification and activation of lysosomal enzymes that can result in DENV degradation. Reducing or slowing phagosome acidification during ADE could allow DENV to escape lysosomal degradation thus promoting enhanced intracellular survival of DENV, albeit with delayed viral uncoating and fusion. To clarify if phagosome acidification is a critical determinant for viral release and subsequent viral replication, we tested if inhibition of phagosome acidification with the lysosomotropic weak bases, ammonium chloride (NH_4_Cl) and CQ, could result in enhanced infection during ADE.

**Figure 4:**
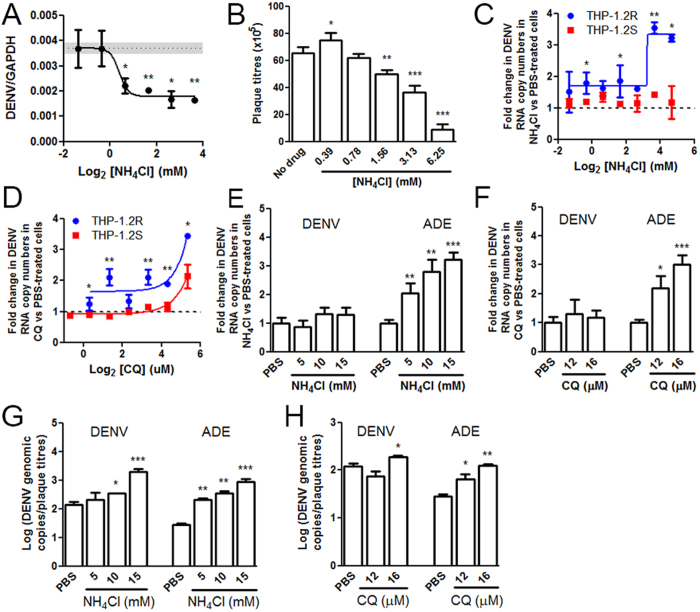
Inhibition of phagosome acidification results in production of immature DENV. (**A**) Viral RNA copy numbers normalized to GAPDH at 20 hpi under DENV-2 conditions in HEK-293T cells treated with ammonium chloride (NH_4_Cl) or PBS (dashed line, shaded areas reflect s.d.). (**B**) Plaque titres at 48 hpi following DENV-2 infection on PBS- or NH_4_Cl-treated HEK-293T cells. (**C**,**D**) Fold change in DENV RNA copy numbers at 20 hpi under ADE conditions in THP-1.2R and THP-1.2S pre-treated with NH_4_Cl (**C**) or chloroquine (CQ) (**D**), relative to cells pre-treated with PBS. (**E**,**F**) Fold change in DENV RNA copy numbers at 16 hpi in primary monocytes pre-treated with NH_4_Cl (**E**) or CQ (**F**), relative to primary monocytes pre-treated with PBS. (**G**,**H**) Ratio of DENV-2 genomic copies to infectious plaque titres at 48 hpi in primary monocytes pre-treated with NH_4_Cl (**G**) or CQ (**H**). Data expressed as mean ± s.d. from three independent experiments. ***P < 0.0001, **P < 0.01, *P < 0.05.

We first verified that we could reproduce the inhibition of both viral replication (Fig. 4A) and infectious DENV titres (Fig. 4B) following DENV infection in HEK-293T cells pre-treated with NH_4_Cl, with no significant increase in cellular cytotoxicity (Supplementary Fig. S3). In contrast, pre-treatment of THP-1 subclones with either NH_4_Cl or CQ led to increased viral replication when infected under ADE condition, with a larger magnitude observed in THP-1.2R (Fig. 4C,D, Supplementary Fig. S4). In contrast, viral replication in THP-1 subclones infected under DENV only condition was not significantly reduced with either CQ or NH_4_Cl pre-treatment (Supplementary Fig. S4).

Pre-treatment of primary monocytes with either CQ or NH_4_Cl before infection also led to increased viral replication, with a larger fold increase under ADE compared to DENV only conditions (Fig. 4E,F, Supplementary Fig. S4). The increased viral replication following CQ or NH_4_Cl pre-treatment consequently resulted in increased production of new viral progenies in the culture supernatant during ADE (Supplementary Fig. S4). Although these virions had reduced infectivity (Fig. 4G,H) due to the expected reduction in viral maturation (Supplementary Fig. S4) from reduced acidification-dependent furin activity, these immature viruses could nonetheless be pathologically important. The abundant presence of non-neutralizing anti-prM antibodies in patients with secondary DENV infection^22^ could render these immature virions infectious again, through ADE^23^.

## Discussion

In both professional and non-professional phagocytes, cellular compartmentalization is utilized as a means of self-defence against human pathogens. Compartment borders restrict pathogen access to the cytosol, and the expression of pattern recognition receptors and danger receptors aid pathogen detection and elimination^8^. Determining how DENV is compartmentalized, depending on the mode of entry, is thus a hitherto unexplored investigation that could provide unique insights into the pathogenesis of dengue.

Plasma membrane receptor signaling during virus uptake can modulate DENV compartmentalization and the outcome of infection. That DENV is destined for phagolysosomal degradation following FcγR-mediated uptake during ADE, is consistent with previous findings that phagosomes containing antibody-opsonized beads matured faster into phagolysosomes, compared to uncoated beads^24^. Furthermore, both *Mycobacterium tuberculosis* (Mtb) and *Helicobacter pylori* opsonized with IgG resulted in more rapid phagosome maturation and were more efficiently destroyed than non-opsonized bacterial particles^25^,^26^. The findings of our study add to this body of knowledge. During ADE, antibody-opsonized DENV co-ligates the ITIM-bearing LILRB1, which signals through SHP-1 to dephosphorylate Syk. Reduced Syk signaling not only results in suppressed ISG induction^6^, it also downregulates phagosome acidification, permitting DENV to escape lysosomal degradation. SHP-1 is thought to inhibit phagosome acidification by acting on membrane fusion during phagosome trafficking^19^. Defects in phagosome acidification have also been observed in SHP-1 deficient mouse macrophages^27^. Our findings thus agree with recent studies that have demonstrated increased viral fusion during ADE, which leads to enhanced production of infectious DENV titres^28^.

Besides DENV, other human pathogens are also known to subvert phagosome acidification for replicative survival. For example, Mtb expresses the bacterial protein tyrosine phosphatase PtpA, which dephosphorylates vacuolar protein sorting 33B. This inhibits recruitment of vacuolar-type H^+^-ATPase (V-ATPase) proton pumps to the phagosome, and inhibits phagosome acidification during Mtb infection^19^. Likewise, *Leishmania donovani* expresses the glycolipid lipophosphoglycan, which inserts into phagosome membranes and inhibits the recruitment of cathepsin D and V-ATPase, which are critical for phagosome acidification^29^. Without expressing proteins that specifically modify phagosome acidification, DENV indirectly alters the phagosome environment by co-ligation of LILRB1 during antibody-dependent infection.

As rapid phagosome acidification for lysosomal protein activation is a key early defense mechanism against infective organisms in the phagosomal cargo, therapeutic approaches that rely on raising intracellular pH should be considered cautiously. We detected increased magnitude of viral replication in both THP-1 subclones and primary monocytes that were pre-treated with CQ or NH_4_Cl during infection under DENV and ADE conditions. Consistent with our findings, the infectivity of both human immunodeficiency virus type 1 and severe acute respiratory syndrome coronavirus were also raised following pre-treatment of lysosomotropic drugs on host cells, as the virus was able to escape lysosomal degradation and enter host cells via a separate endocytic pathway^30^,^31^.

Our findings also potentially explain, at least in part, why CQ failed to show therapeutic efficacy in a proof-of-concept clinical trial in dengue patients^32^. Although CQ and NH_4_Cl treatment-related increase in viral RNA replication is more abundantly secreted as immature DENVs, these could nonetheless be detrimental in patients experiencing secondary dengue infections, which formed the majority of the trial subjects^32^. Prior DENV infection in such patients would likely have produced abundant cross-reactive anti-prM antibodies^22^, which would bind immature DENV particles to enable entry into FcγR-bearing cells where subsequent furin activity renders them infectious^23^.

Collectively, our findings suggest that DENV adopts a “*festina lente*” model to escape phagolysosomal degradation, ensuring its intracellular survival during ADE. Although reduced or slowed phagosome acidification during ADE may delay viral fusion and uncoating, it is probably a necessary trade-off that ultimately permits DENV to escape lysosomal degradation in favor of intracellular survival.

## Methods

### Cells and viruses

THP-1.2R and THP-1.2S were sub-cloned from THP-1 by limiting dilution^6^. Primary monocytes were isolated from principal investigator and cultured as described previously^33^. DENV-2 (ST) is a clinical isolate from the Singapore General Hospital. The method for DiD labeling of DENV was as previously described^33^. Dual labeling of DENV with pHrodo and Alexa Fluor 488 is detailed in Supplementary Methods.

### Virus infection

Endotoxin-free (LAL Chromogenic Endotoxin Quantitation Kit, Pierce) 3H5 chimeric human/mouse IgG1 antibodies were constructed as previously described^34^. DENV was incubated with media or humanized 3H5 antibody (0.391 μg/ml) for 1hr at 37 °C before adding to cells at moi of 10. In some experiments, DENV was dual labeled with pHrodo Red and Alexa Fluor 488 or DiD before use for infection (Supplementary Methods). For drug assays, cells were pre-treated with chloroquine (Sigma-Aldrich) or ammonium chloride (Sigma-Aldrich) 1hr before infection, or sodium stibogluconate (Santa Cruz Biotech) 6 hrs before infection. Cell viability was assessed using CellTiter 96^®^ AQueous One Solution Cell Proliferation Assay (Promega) according to manufacturer’s protocol. Subsequently, virus replication was assessed using qPCR at indicated time points and plaque assay at 48 hrs or 72 hrs post-infection. Protein and protein phosphorylation levels were assessed using Western blots and analyzed with ImageJ.

### Isolation of DENV endocytic vesicles

Isolation of DENV endocytic vesicles was performed using a protocol previously used for the isolation of latex bead phagosomes^14^. Briefly, DENV was incubated with media or h3H5 (0.391 μg/ml) for 1 hr at 37 °C before adding to THP-1.2R and THP-1.2S (moi 10). Cells were incubated with DENV immune complex at 4 °C for 20 min before transferring to 37 °C for 2hrs. Cells were washed with PBS, homogenized on ice in 25 strokes using a 30-G syringe and DENV endocytic vesicles were isolated on either a step or continuous sucrose gradient. 250 μl fractions from the continuous sucrose gradient were collected and subjected to viral RNA extraction using QiaAmp Viral RNA Mini kit (Qiagen). Fractions at the interfaces of step sucrose gradients were collected, with 140 μl subjected to viral RNA extraction. DENV endocytic vesicles were resuspended in 10 ml cold PBS containing protease inhibitors and washed by ultracentrifugation. The protein pellet was resuspended in 100 μl lysis buffer (1% Nonidet P-40, 150 mM NaCl, 50 mM Tris, pH 8.0) in the presence of protease and phosphatase inhibitors (Sigma). Protein concentration of DENV endocytic vesicles was determined using Pierce BCA Protein Assay Kit (Pierce) and endocytic vesicle proteins (3 μg) separated on a SDS-PAGE gel for immunoblotting.

### Nanoparticle tracking analysis (NTA)

Characterization of DENV endocytic vesicles was performed with Nanosight LM10, customized with a 692 nm bandpass filter for use under fluorescence mode. Washed DENV endocytic vesicles were resuspended at an appropriate dilution in HNE buffer before injection into the LM unit with a 1 ml syringe. NTA 2.3 analytical software was used to capture and analyze data. For each sample, 5 videos of 90 s duration were recorded, with a 5 s delay between recordings and chamber temperature recorded at the end of each video.

### Assessing phagosome acidification with pHrodo/AF488-labeled DENV

pHrodo/AF488-labelled DENV were incubated with media or h3H5 (0.391 μg/ml) for 1hr at 37 °C before adding to THP-1 cells (moi 10). Cells were synchronized on ice for 20 min, followed by 2 hrs infection at 37 °C and fixed with 3% PFA. Confocal immunofluorescence was used to assess localization of antibody–virus complexes and fluorescence intensities of pHrodo and AF488 on DENV in the cell. Detailed description is provided in Supplementary Methods.

### Statistical Analysis

All experiments were conducted with at least 3 biological replicates and repeated at least twice. Mann-Whitney test was used to compare differences in phagosome acidification. In other experiments, to compare between any two means, two-tailed unpaired Student t test was performed using GraphPad Prism v5.0 (*P* < 0.05).

## Additional Information

**How to cite this article:** Ong, E. Z. *et al*. Dengue virus compartmentalization during antibody-enhanced infection. *Sci. Rep.* **7**, 40923; doi: 10.1038/srep40923 (2017).

**Publisher's note:** Springer Nature remains neutral with regard to jurisdictional claims in published maps and institutional affiliations.

## Supplementary Material

Supplementary Information
