# Supplementary material for: Dengue virus compartmentalization during antibody-enhanced infection
Source: Sci Rep. 2017 Jan 13;7:40923. doi: 10.1038/srep40923 (PMC5234037; doi:10.1038/srep40923)
Supplement: Supplementary Information [file srep40923-s1.pdf]

Supplementary Information file

Dengue virus compartmentalization during antibody-enhanced infection

Eugenia Z. Ong[1,2], Summer L. Zhang[2], Hwee Cheng Tan[2], Esther S. Gan[2], Kuan Rong Chan[2], Eng Eong Ooi[2,3,4,5,*]

[1]Experimental Therapeutics Centre, Agency for Science, Technology and Research (A*STAR), Singapore 138669

[2]Program in Emerging Infectious Diseases, Duke-NUS Medical School, Singapore 169857

[3]Department of Microbiology and Immunology, National University of Singapore, 8 Medical Drive, Block MD4, Singapore 117545

[4]Saw Swee Hock School of Public Health, National University of Singapore, 12 Science Drive 2, Singapore 117597

[5]Singapore MIT Alliance Research and Technology, Infectious Diseases Interdisciplinary Research Group, CREATE Campus, Singapore 138602

* Correspondence to: Eng Eong Ooi BMBS, PhD, Program in Emerging Infectious Disease, Duke-NUS Graduate Medical School, Tel: +65 65168594, Fax: +65 62212529

Email: engeong.ooi@duke-nus.edu.sg

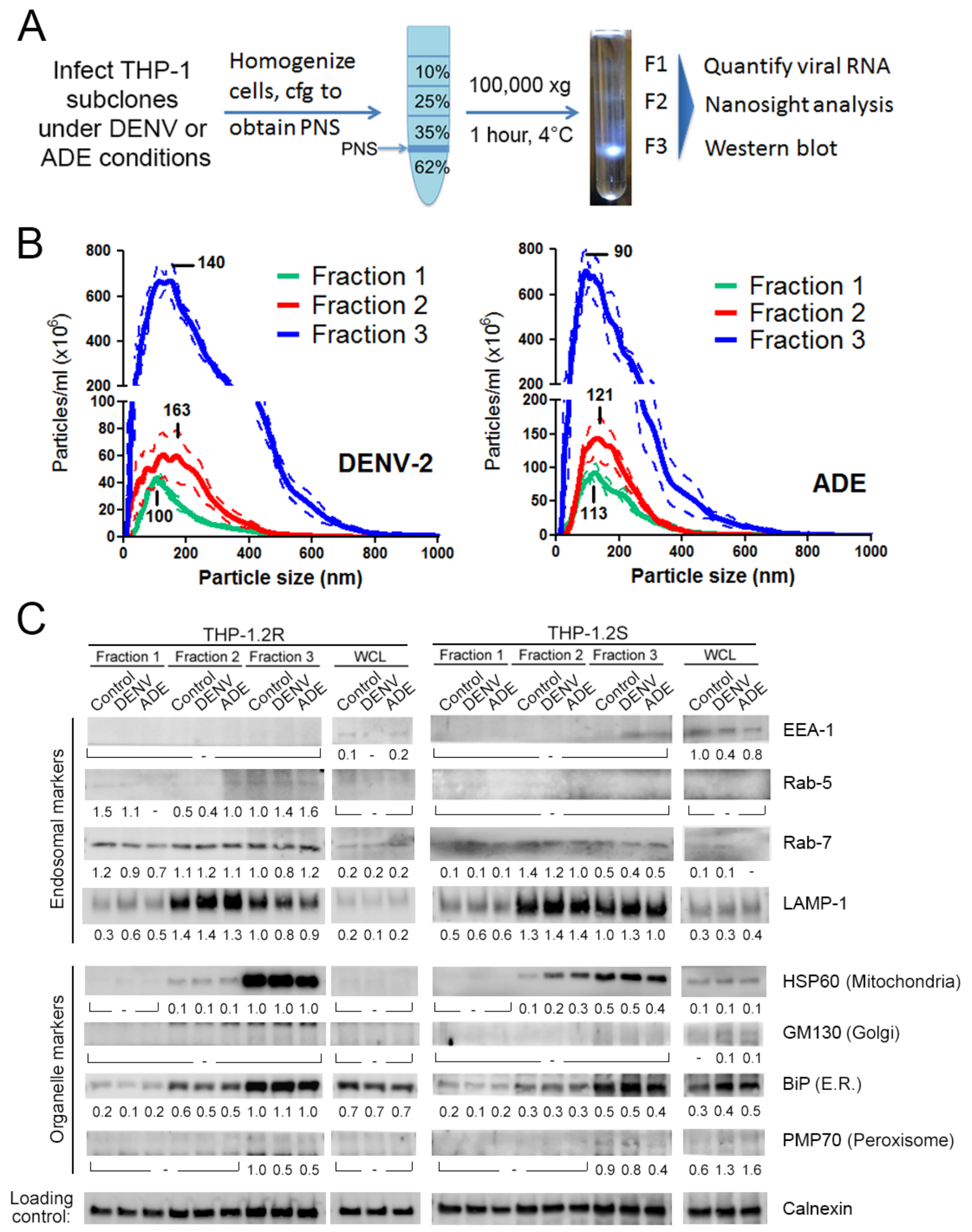

Supplementary Figure S1. Isolation of DENV endocytic vesicles on a step sucrose gradient. (A) Workflow for isolation of DENV endocytic vesicles on a step sucrose gradient. (B) Concentration and modal size (numbers indicated) of all nanoparticles from fractions collected from THP-1.2S at 2hpi under DENV-2 or ADE conditions. Solid line indicates mean value, and dotted lines indicate s.d. (C) Western blot analysis of subcellular fractions, probed for endosomal markers and organelle markers. Step sucrose gradient purified fractions were collected from THP-1.2R or THP-1.2S infected under DENV only or ADE conditions, or uninfected control cells. Whole cell lysate (WCL) was obtained before ultracentrifugation of cell lysates to obtain subcellular fractions. Numbers below Western blots indicate protein levels relative to calnexin, while "-" indicates protein is not expressed. Data expressed as mean ± s.d. from at least two independent experiments.

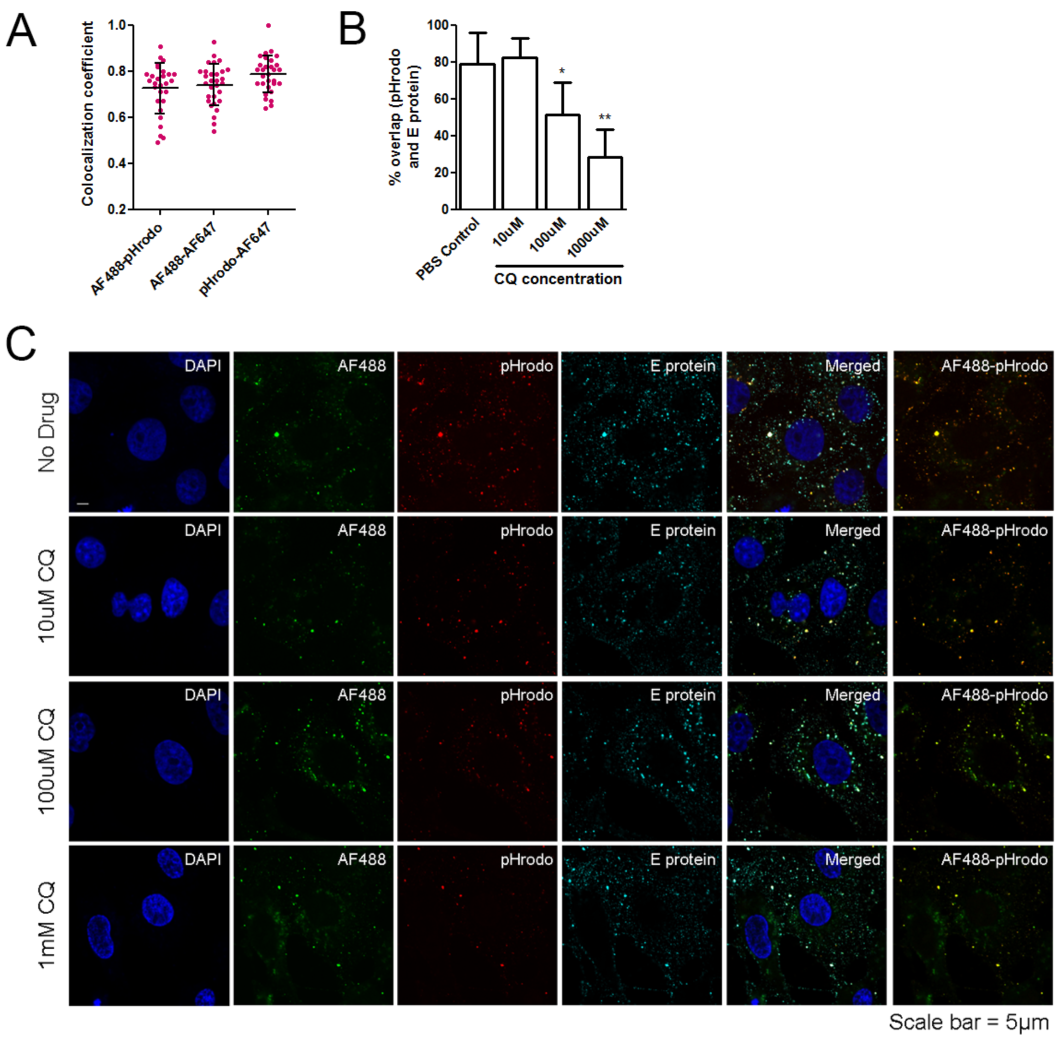

Supplementary Figure S2. Dual labelling of DENV with pHrodo and Alexa Fluor 488 (AF488). (A) Vero cells were infected with pHrodo/AF488-labelled DENV for 15min at 37°C. Thereafter, cells were fixed and labelled with anti-E antibody, and examined for colocalization of E protein (AF647, cyan) with AF488 (green) and pHrodo (red) labelling. Quantification of colocalization for pHrodo and AF488 with DENV E protein (AF647) was determined using confocal microscopy. Each dot represents a single virus particle, line reflects mean value and error bars indicate s.d. (B) pHrodo fluorescence intensity decreases as pH becomes less acidic. Percent overlap for pHrodo and E protein (cyan) in PBS- or chloroquine (CQ)-treated Vero cells, as determined using spot analysis performed on Imaris. (C) Co-localization of DENV labelled with AF488 (green) and pHrodo (red) with E protein (cyan) in Vero cells treated with varying concentrations of CQ at 15min post-infection. Data expressed as mean ± s.d. from at least two independent experiments. ** $P < 0.01$, * $P < 0.05$.

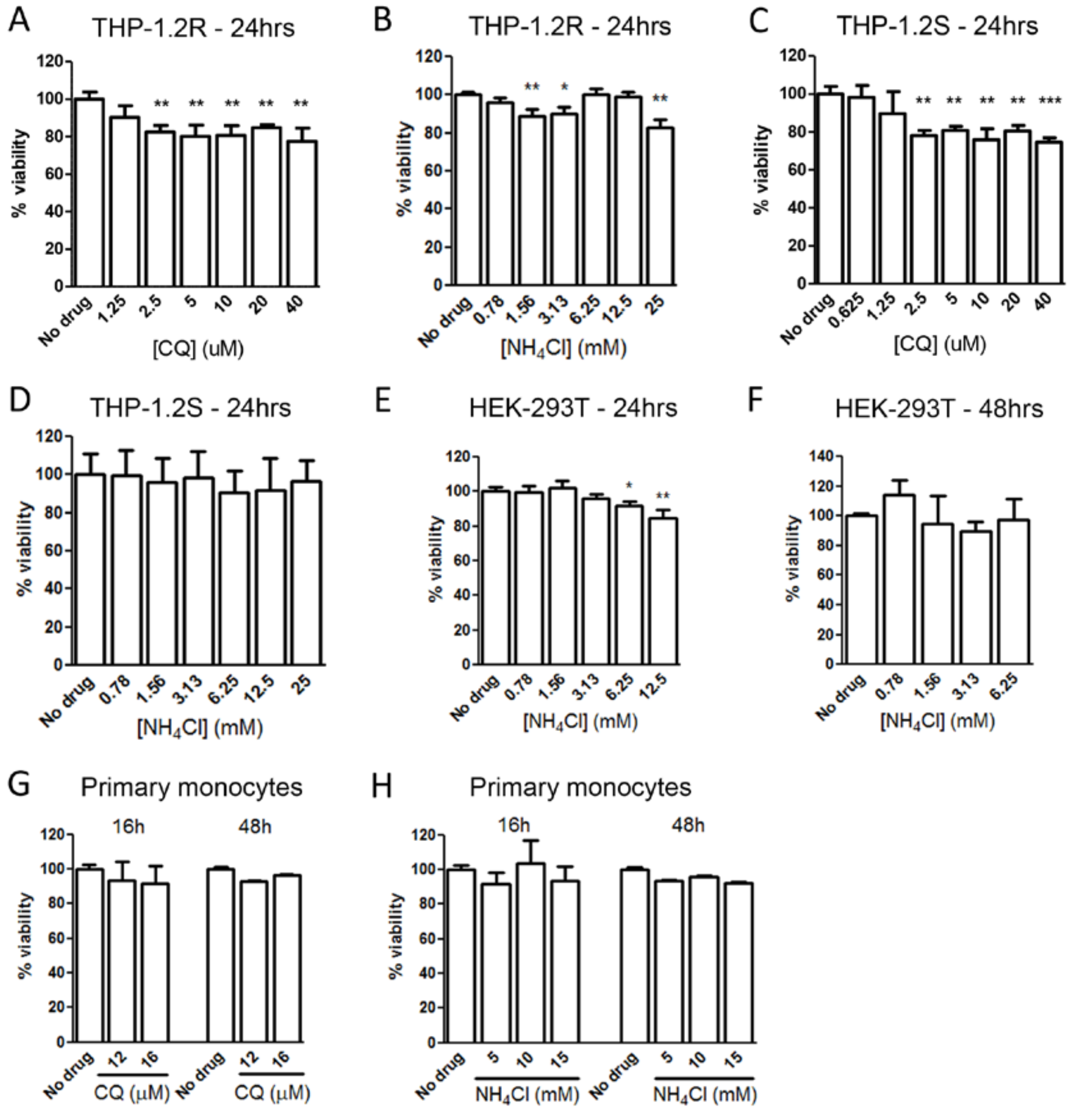

Supplementary Figure S3. Cell viability following drug treatment. (A-H) Cell viability after drug treatments at time points indicated, on THP-1.2R (A-B), THP-1.2S (C-D), HEK-293T (E-F), and primary monocytes (G-H). Cell viability was assessed using CellTiter 96® Aqueous One Solution Cell Proliferation Assay (MTS, Promega) according to manufacturer's protocol. Experiments were performed in triplicates and data expressed as mean ± s.d. *** $P < 0.0001$, ** $P < 0.01$, * $P < 0.05$.

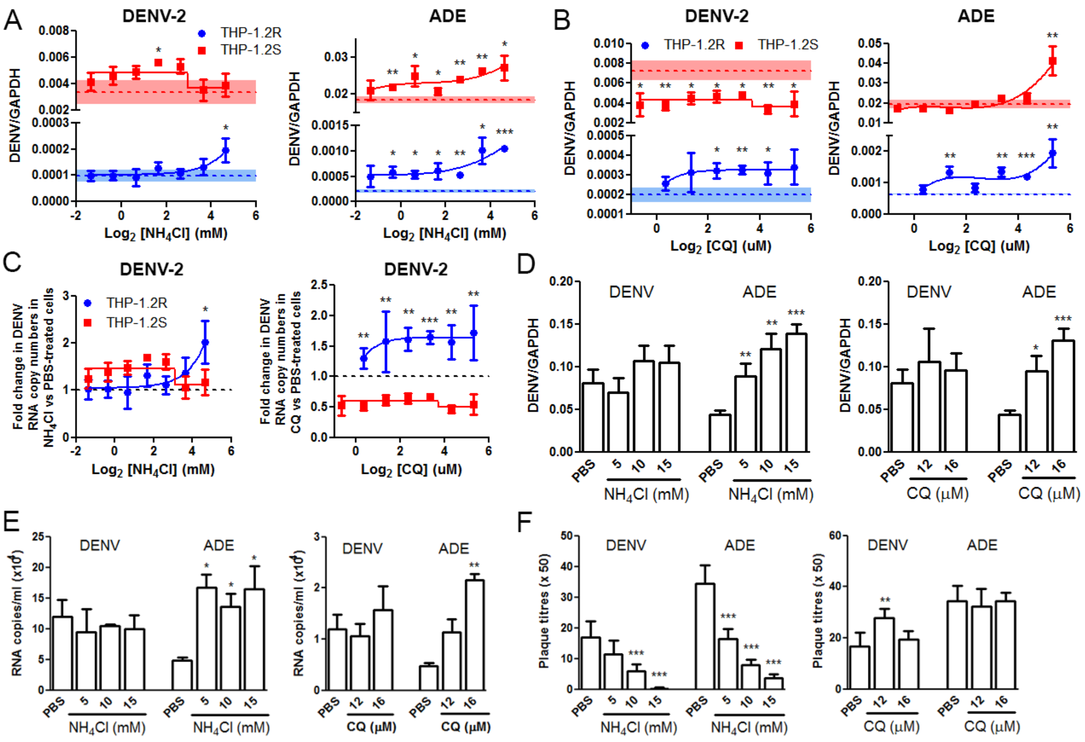

Supplementary Figure S4. Inhibition of phagosome acidification with chloroquine (CQ) or ammonium chloride (NH_4Cl) results in production of immature DENV. (A-B) Viral RNA copy numbers normalized to GAPDH at 20hpi under DENV-2 and ADE conditions in THP-1.2R and THP-1.2S pre-treated with PBS (dashed lines, shaded areas reflect s.d.), NH_4Cl (A) or CQ (B). (C) Fold change in DENV RNA copy numbers during DENV-2 infection in cells pre-treated with NH_4Cl or CQ, relative to PBS control. (D) Viral RNA copy numbers normalized to GAPDH at 16hpi under DENV-2 or ADE conditions in primary monocytes pre-treated with NH_4Cl, CQ or PBS. (E-F) Viral RNA copy numbers in supernatant (E) and plaque titers (F) following DENV-2 or ADE infection on primary monocytes pre-treated with PBS, NH_4Cl or CQ at 48hpi. Data expressed as mean ± s.d. from three independent experiments. *** $P < 0.0001$, ** $P < 0.01$, * $P < 0.05$.

Supplementary Methods

Cells and viruses

THP-1 subclones, THP-1.2R (ADE-resistant) and THP-1.2S (ADE-susceptible) were subcloned from THP-1 by limiting dilution [6]. Primary monocytes were isolated from principal investigator and cultured as described previously [34]. Cell lines used were negative for mycoplasma contamination (Mycoalert, Lonza). DENV-2 (ST) is a clinical isolate from the Singapore General Hospital. Viruses were propagated in the Vero cell line, harvested 96h post infection and purified through 30% sucrose. Virus pellets resuspended in HNE buffer were stored at −80 °C until use.

Virus infection

Endotoxin-free (LAL Chromogenic Endotoxin Quantitation Kit, Pierce) 3H5 chimeric human/mouse IgG1 antibodies were constructed as previously described [35]. DENV was incubated with media or humanized 3H5 antibody for 1hr at 37˚C before adding to cells at moi of 10. Virus replication was assessed at indicated time-points using qPCR at indicated time points. Cells were washed thrice in PBS, followed by RNA extraction using RNAeasy kit (Qiagen), cDNA synthesis (Invitrogen) and real-time qPCR (Roche) according to manufacturer's protocol. DENV primers used were:
DEN-F 5'-TTGAGTAAACYRTGCTGCCTGTAGCTC,
DEN-R 5'-GAGACAGCAGGATCTCTGGTCTYTC.
Primers used for GAPDH were from Origene, and RNA levels were measured relative to GAPDH. At 48hpi or 72hpi, virus in the culture supernatant was quantified with plaque assay [34].

Dual labelling of DENV with pHrodo Red and Alexa Fluor 488

Labelling buffer (0.2M sodium bicarbonate buffer, pH 8.3) was freshly prepared and filter sterilized with 0.2µm syringe filters prior to DENV labelling. Purified DENV was diluted to approximately $3x10^8$ PFU in 1ml of labelling buffer. Both lyophilized Alexa Fluor 488 (AF488) succinimidyl esters and pHrodo Red succinimidyl esters were reconstituted to 2mM in labelling buffer or DMSO respectively, immediately prior to the labelling reaction. pHrodo Red dye was added to the diluted virus at a final concentration of 100µM, while stirring gently with the pipette tip. The labelling reaction mix was incubated at room temperature for 30min in the dark and mixed by gentle inversions every 15min. Next, AF488 dye was added to the diluted virus at a final concentration of 100µM, while stirring gently with the pipette tip. The labelling reaction mix was incubated at room temperature for an additional 1hr in the dark and mixed by gentle inversions every 15min. Excess dye was removed by gel filtration on a PD-10 column (GE Healthcare). Briefly, the column was equilibrated with 25ml of HNE buffer (5mM Hepes, 150mM NaCl, 0.1mM EDTA, pH 7.4) before use. The labelled virus was applied to the top of the column and collection of flow-through began once the labelled virus entered the matrix. The first 3.25ml of flow-through was discarded, while the following 2ml of labelled virus fraction was collected. The pHrodo/AF488-labelled DENV was stored in 100µL aliquots at −80 °C, away from light source, retitrated by plaque assay, and tested for fluorescence using immunofluorescence assay on Vero cells before use in experiments.

DiD Labeling of dengue virus

For DiD (1, 1′-dioctadecyl-3, 3, 3′, 3′-tetramethylindodicarbocyanine, 4 chlorobenzenesulfonate salt) labeling, approximately $3x10^8$ PFU DENV was mixed with 800 nmol of DiD (Invitrogen) in DMSO (final DMSO concentration <2.5%). After 30 min, free DiD was removed by gel filtration on a Sephadex G-25 column (Amersham Biosciences) equilibrated in HNE buffer. DiD-labeled DENV was stored at 4 °C and used within 24hrs.

Immunofluorescence of virus infection on Vero cells

Equal volumes of pHrodo/AF488-labelled DENV were incubated with Vero cells plated on coverslips for 15min at 37°C, washed, fixed with 3% paraformaldehyde (PFA) and permeabilized with 0.1% saponin. In some experiments, Vero cells were pre-treated with chloroquine at indicated doses for 30min at 37°C before infection with pHrodo/AF488-labelled DENV. The cells were then incubated for 1 hr with anti-E protein h3H5 monoclonal antibody, at room temperature. The cells were washed three times in PBS, followed by incubation with AF647 anti-human IgG antibody for 45min at room temperature. Cells were then washed three times in PBS, rinsed once in deionised water and mounted on to glass slides with Mowiol 4-88. Cells were visualized at 63x magnification on a Zeiss LSM710 confocal microscope and co-localization coefficients were calculated using Zeiss ZEN2011 program.

Spot analysis was performed by Imaris to identify $AF647^+$ (E protein) and $pHrodo^+$ particles in each image. Particles were filtered by size and visually confirmed. Images were exported in individual colours for processing in Adobe Photoshop CS6 version 13, which involved adjustment of the contrast on the images for clarity. Images in individual colours were then merged using ImageJ.

Assessing phagosomal acidification with pHrodo/AF488-labelled DENV

pHrodo/AF488-labelled DENV was incubated with maintenance media or sub-neutralizing concentrations of h3H5 (0.391µg/ml) for 1hr at 37°C before adding to THP-1.2R, THP-1.2S or primary monocytes (moi 10). Cells were synchronized on ice for 20min, followed by 2hrs infection at 37°C and fixed with 3% PFA for 30min at room temperature. Cells were subjected to cytospin at 800rpm for 3min. After washing with PBS, infected cells were permeabilized with 0.1% saponin for 30min and incubated with anti-E protein or anti-LAMP1 antibody at 4°C overnight. Cells were washed with PBS, and incubated with AF647 anti-human IgG antibody or AF647 anti-mouse IgG antibody for 45min at room temperature. Cells were then washed three times in PBS, rinsed once in deionised water and mounted on to glass slides with Mowiol 4-88. Cells were visualized at 63x magnification on a Zeiss LSM710 confocal microscope and fluorescence intensities of pHrodo and AF488 were determined using ImageJ. Briefly, background fluorescence was first subtracted (rolling ball radius, 50 pixels). Individual virus particles were identified as a region of interest (ROI) and fluorescence intensities of pHrodo and AF488 were determined using the Multimeasure function in ImageJ. Fluorescence intensities of DENV phagosomes in 25 to 30 randomly selected cells were calculated. Only DENV phagosomes with AF488 or pHrodo fluorescence intensity more than 20 were included in the analysis. To assess levels of phagosome acidification, the ratio of fluorescence intensity for pHrodo to AF488 was calculated for each phagosome.

Isolation of DENV endocytic vesicles on a step sucrose gradient

Isolation of DENV endocytic vesicles was performed using a protocol previously used for the isolation of latex bead phagosomes [14]. Briefly, DENV was incubated with media or h3H5 (0.39µg/ml) for 1hr at 37˚C before adding to cells at indicated moi. THP-1.2R and THP-1.2S were incubated with DENV immune complex at 4°C for 20min before transferring to 37°C for 2hrs. After 2hrs, cells were washed 3 times with PBS before resuspending in homogenization buffer (8.55% (w/v) sucrose in HNE buffer, 3mM imidazole, 1% protease inhibitor solution, pH 7.4). To isolate DENV endocytic vesicles, cells were homogenized on ice in 25 strokes using a 30-G syringe. The homogenate was spun down at 2000 rpm for 5min to remove cell debris and nuclei. The supernatant was collected and mixed with an equivalent volume of 62% (w/v) sucrose to bring it to a final concentration of 40% sucrose. DENV endocytic vesicles were then isolated on a discontinuous sucrose gradient, which was prepared as follows: 3ml 62% sucrose, 2ml of 40% sucrose phagosome suspension, 2ml of 35% sucrose, 2ml of 25% sucrose and 2ml of 10% sucrose. Centrifugation was performed at 4°C in a swinging bucket rotor (SW41; Beckman Instruments) for 1h at 100, 000 x g. Fractions at the interfaces of step sucrose gradients were collected, with 140µl subjected to viral RNA extraction using QiaAmp Viral RNA Mini kit (Qiagen). The collected fractions were resuspended in 10ml cold PBS containing protease inhibitors and pelleted by ultracentrifugation (40,000 x g, 30min) at 4°C in an SW41 rotor. Supernatant was removed and the protein pellet was resuspended in 100µl lysis buffer (1% Nonidet P-40, 150mM NaCl, 50mM Tris, pH 8.0) in the presence of protease and phosphatase inhibitors (Sigma). The protein concentration of DENV endocytic vesicles was determined using Pierce BCA Protein Assay Kit (Pierce). Endocytic vesicle proteins (3µg) were separated on a SDS-PAGE gel for Western blot analysis. For nanoparticle tracking analysis (NTA), washed DENV endocytic vesicles were resuspended at an appropriate dilution in HNE buffer before injection into the LM unit with a 1ml syringe.

Isolation of DENV-containing endocytic vesicles on a continuous sucrose gradient

For isolation of DENV endocytic vesicles on a continuous sucrose gradient, sucrose gradient was first formed by careful layering of 10% to 60% sucrose in 10% increments, starting with the densest at the bottom. The gradient was allowed to linearize overnight at 4°C. Cell homogenate was layered above the continuous sucrose gradient for ultracentrifugation. After ultracentrifugation, 250µl fractions were collected and subjected to viral RNA extraction using QiaAmp Viral RNA Mini kit (Qiagen).

Nanoparticle tracking analysis (NTA)

The Nanosight LM10 utilizes a single-mode red laser diode with illumination of 638nm wavelength and laser power of 25mW to visualize nanoparticles ranging from 10 to 1000nm in size. It was also customized with a 692nm bandpass filter for use under fluorescence mode. The red laser illuminates DiD-labelled nanoparticles in suspension diluted to between 10^7 and 10^9 particles per ml in light scatter mode or fluorescence mode. A high sensitivity scientific CMOS camera tracks individual particles moving under Brownian motion and particle distribution and size, as calculated using the Stokes-Einstein equation, was analyzed with the NTA 2.3 analytical software (Nanosight). The camera settings (shutter, gain, detection threshold and sensitivity) were optimized for individual samples. For each sample, 5 videos of 90s duration were recorded, with a 5s delay between recordings and chamber temperature recorded at the end of each video.

Immunoblotting

DENV endocytic vesicles (3µg) were separated on a SDS-PAGE gel for Western blot analysis and probed with primary antibody, followed by HRP-conjugated anti-mouse (1:1000, Dako P0447) or anti-rabbit (1:3000, Abcam ab6721) antiserum. Primary antibodies for LILRB1 (1:500, Abcam ab67532), SHP-1 (1:500, Abcam ab2020), pSHP-1 (1:500, Abcam ab51171), GAPDH (1:3000, Abcam ab8245), Rab-5 (1:1000, Abcam ab13253), Rab-7 (1:1000, Abcam ab50533), LAMP-1 (1:1000, eBioscience 611043), Cathepsin D (1:500, Abcam ab6313), EEA-1 (1:1000, Santa Cruz sc33585), BiP (1:1000, Abcam ab21685), calnexin (1:1000, Abcam ab22595), PMP70 (1:500, Abcam ab3421), GM130 (1:10,000, Abcam ab52649) and HSP60 (1:20,000, Abcam ab59457) were used. Thereafter, blots were developed by enhanced chemiluminescence detection reagents (Amersham).

iTRAQ labeling and SCX chromatography

iTRAQ analysis on DENV endocytic vesicles were performed at Beijing Genomics Institute. Protein concentration was determined using the Pierce BCA Protein Assay kit. Protein samples were digested using Trypsin Gold (Promega) at a ratio of 1:20 for 12hrs at 37°C, and labeled using iTRAQ Reagent 8-plex kits (AB Sciex) according to manufacturer's protocol. Samples were labeled with iTRAQ tags as follows: uninfected THP-1.2R (113), uninfected THP-1.2S (118), DENV infected THP-1.2R (117), DENV infected THP-1.2S (115), ADE infected THP-1.2R (114 and 119) and ADE infected THP-1.2S (116 and 121). Labeled peptides were then fractionated using strong cation exchange (SCX) chromatography on the Shimadzu LC-20AB HPLC Pump system. Peptides were first resuspended in 4ml buffer A (25mM NaH_2PO_4 in 25% ACN, pH 2.7) and loaded on an Ultramex SCX column (4.6 x 250mm). Peptides were eluted at a flow rate of 1ml/min with a gradient of buffer A for 10 min, 5-35% buffer B (25 mM NaH_2PO_4, 1M KCl in 25% ACN, pH 2.7) for 11 min, 35-80% buffer B for 1 min. The system was then maintained in 80% buffer B for 3 min before equilibrating with buffer A for 10 min prior to the next injection. Elution was monitored by measuring absorbance at 214 nm, and fractions were collected every 1 min. The eluted peptides were pooled as 12 fractions, desalted and vacuum-dried before LC-ESI MS/MS analysis.

LC and MS

Each fraction was resuspended in buffer A (2% ACN, 0.1% FA) and centrifuged at 20,000 x g for 10min. 10ul supernatant was loaded on a Shimadzu LC-20AD nano-HPLC at a flow rate of 15ul/min for 4 min. Peptides were eluted over a 44 min gradient, as follows: 400 nL/min starting from 2 to 35% solvent B (98%ACN, 0.1%FA), followed by a 2min linear gradient to 80%, and maintenance at 80% solvent B for 4 min, and returning to 2% solvent B in 1 min. Data acquisition was performed using an AB Sciex TripleTOF 5600 System. Data was acquired using an ion spray voltage of 2.5 kV, curtain gas of 30 PSI, nebulizer gas of 15 PSI, and an interface heater temperature of 150 °C. The MS was operated with a RP greater than or equal to 30,000 FWHM for TOF MS scans. For information-dependent data acquisition (IDA), survey scans were acquired in 250 ms and up to 30 product ion scans were collected if exceeding a threshold of 120 counts per second (counts/s) and with a 2+ to 5+ charge-state. Total cycle time was fixed to 3.3s. The Q2 transmission window was 100Da for 100%. Four time bins were summed for each scan at a pulser frequency value of 11 kHz through monitoring of the 40 GHz multichannel TDC detector with 4-anode/channel detection. A sweeping collision energy setting of 35±5 eV with adjust rolling collision energy was applied to all precursor ions for collision-induced dissociation. Dynamic exclusion was set for 1/2 of peak width (18 s), and then the precursor was refreshed off the exclusion list.

iTRAQ protein identification and analysis
Identification and quantitation of proteins was performed by searching the resultant peak lists against the Uniprot human database (143,397 sequences) using the search engine Mascot MS/MS Ions Search (v2.3.02). The search parameters were as follows: enzyme specificity set to trypsin with one missed cleavage; MS/MS fragment ion mass tolerance of ±0.1 Da; fixed modifications of carbamidomethylation at Cys and iTRAQ 8 plex at Lys and the N-terminal amino group of peptides; variable modifications of oxidation at Met, iTRAQ 8 plex at Tyr, and glutamine as pyroglutamic acid; peptide change was set at Mr and monoisotopic mass was chosen. Differentially expressed proteins were determined using a 1.2-fold cutoff, with a p-value <0.05. Data analysis was performed using the Molecular Signatures database that accompanies the Gene Set Enrichment Analysis package (Broad Institute). Lists of differentially regulated proteins were analysed with KEGG gene sets to identify enriched pathways.

Drug assays
$2x10^5$ cells/ml of THP-1.2R, THP-1.2S or HEK-293T were pre-treated for 1hr with chloroquine, ammonium chloride or PBS control before adding DENV-2 or h3H5-opsonized DENV-2. $2x10^5$ cells/ml of primary monocytes were pre-treated for 1hr with chloroquine, ammonium chloride or PBS control in flat-bottom microtiter plates, before adding DENV-2 or h3H5-opsonized DENV-2. For sodium stibogluconate (0.55mM) treatment, $2x10^5$ cells/ml of primary monocytes were treated for 6hrs before pHrodo/AF488-labelled DENV, opsonized or not with h3H5, were added. Cell viability was assessed using CellTiter 96® Aqueous One Solution Cell Proliferation Assay (MTS, Promega) according to manufacturer's protocol.

Receptor blocking
$2x10^5$ cells/ml of primary monocytes were pre-treated with 10µg/ml of anti-LILRB1 or isotype control antibody for 1hr at 4ºC. Subsequently, cells were washed once with maintenance media before adding pHrodo/AF488-labelled DENV opsonized or not with h3H5 (0.391µg/ml).

siRNA transfection and overexpression
Knockdown and overexpression studies were performed as previously described [34]. siRNA targeting LILRB1 (SABio) were used while overexpression studies were performed with either empty plasmid or plasmid encoding LILRB1 (Origene).

Statistical analysis
All experiments were conducted with at least 3 biological replicates and repeated at least twice. Mann-Whitney test was used to compare differences in phagosome acidification. In other experiments, to compare between any two means, two-tailed unpaired Student t test was performed using GraphPad Prism v5.0 (GraphPad Software Inc) (P<0.05).
